# We Have Spent Time, Money, and Effort Making Self-Help Digital Mental Health Interventions: Is Anyone Going to Come to the Party?

**DOI:** 10.2196/58198

**Published:** 2024-09-19

**Authors:** Skye Fitzpatrick, Alexander O Crenshaw, Victoria Donkin, Alexis Collins, Angela Xiang, Elizabeth A Earle, Kamya Goenka, Sonya Varma, Julianne Bushe, Tara McFadden, Andrea Librado, Candice Monson

**Affiliations:** 1 York University Toronto, ON Canada; 2 Kennesaw State University Kennesaw, GA United States; 3 University of Waterloo Waterloo, ON Canada; 4 Atlas Institute for Veterans and Families Ottawa, ON Canada

**Keywords:** online interventions, self-help, digital interventions, mental health, psychotherapy, intervention desirability

## Abstract

Although efficacious psychotherapies exist, a limited number of mental health care providers and significant demand make their accessibility a fundamental problem. Clinical researchers, funders, and investors alike have converged on self-help digital mental health interventions (self-help DMHIs) as a low-cost, low-burden, and broadly scalable solution to the global mental health burden. Consequently, exorbitant financial and time-based resources have been invested in developing, testing, and disseminating these interventions. However, the public’s assumed desirability for self-help DMHIs by experts has largely proceeded without question. This commentary critically evaluates whether self-help DMHIs can, and will, reach their purported potential as a solution to the public burden of mental illness, with an emphasis on evaluating their real-world desirability. Our review finds that self-help DMHIs are often perceived as less desirable and credible than in-person treatments, with lower usage rates and, perhaps accordingly, clinical trials testing self-help DMHIs suffering from widespread recruitment challenges. We highlight two fundamental challenges that may be interfering with the desirability of, and engagement in, self-help DMHIs: (1) difficulty competing with technology companies that have advantages in resources, marketing, and user experience design (but may not be delivering evidence-based interventions) and (2) difficulty retaining (vs initially attracting) users. We discuss a range of potential solutions, including highlighting self-help DMHIs in public mental health awareness campaigns; public education about evidence-based interventions that can guide consumers to appropriate self-help DMHI selection; increased financial and expert support to clinical researchers for marketing, design, and user experience in self-help DMHI development; increased involvement of stakeholders in the design of self-help DMHIs; and investing in more research on ways to improve retention (versus initial engagement). We suggest that, through these efforts, self-help DMHIs may fully realize their promise for reducing the global burden of mental illness.

## We Have Spent Time, Money, and Effort Making Online Self-Help Interventions: Is Anyone Going to Use Them?

Since their inception, psychotherapy interventions have offered inherently individual-level solutions for what are societally pervasive problems of mental illness. For example, early psychoanalytic interventions were offered to members of the Austrian elite and required exorbitant provider resources (eg, up to 6 to 10 hours per week for 6 months to 3 years) [1]. Given this, it is probably safe to say that “accessible mental health for all” was not the original framework upon which many psychological interventions were built. Although individually beneficial, the inadequacy of psychotherapy in combating the global burden of mental illness is clear; more than 70% of people with mental illness do not receive any mental health intervention [2]. In response to this problem, contemporary interventionists have sought to translate inaccessible psychological interventions for mass consumption. Given their low-cost, low-burden, and broadly scalable potential, one proposed solution that appears particularly promising is self-help digital mental health interventions (self-help DMHIs).

Self-help DMHIs are a class of interventions that are self-directed and delivered over online platforms. They often combine psychoeducational materials (in text, video, or audio format) and explicit practice and implementation of psychotherapeutic concepts in a structured format. They may also leverage web browsers, apps, or other forms of digital technologies (eg, texting) as their mode of delivery. Self-help DMHIs may or may not involve some minimal contact with a paraprofessional (eg, a “coach”) but, crucially, do not involve extensive one-on-one time with a mental health care professional. We argue that, once a mental health care professional becomes involved in delivering an intervention, that intervention is no longer “self-help.” Owing to their perceived potential, researcher, clinician, funder, and investor interest in self-help DMHIs has flourished [3], and decades of work has now been conducted in this arena. Although some experts suggest that self-help DMHIs may represent a scalable mental health solution [2,4-6], it is less clear whether they are actually desirable to the public. As the time, money, and research invested into self-help DMHIs grows, this commentary aims to take stock of what is known about their desirability and consider whether their promise has been (or can be) realized. First, we present a discussion of why self-help DMHIs may be a compelling mental health solution; review the way that they have amassed attention, resources, and investment from multiple sectors; and identify later-revealed problems in their uptake and desirability. Our review of these issues largely centers on a North American context, and we encourage more empirical attention toward whether the financial and empirical interest in self-help DMHIs, as well as difficulties with their uptake, is moderated by region of the world. We then discuss potential reasons for desirability and uptake issues in self-help DMHIs and present possible solutions.

## The Rise (and Potential Fall?) of Self-Help DMHIs: A Party Planning Story

### Let’s Have a Party: Self-Help DMHIs Are the Future (and the Present)!

Self-help DMHIs have long been purported to be a central solution to the joint problems of mental health burden and lack of available evidence-based mental health interventions [2,4-6]. There are several reasons for this optimism. First, self-help DMHIs’ reliance on technology and focus on scalability means that once built, they are relatively low-cost to maintain compared to the number of people they can serve. They may not require the resources of highly educated mental health care professionals or, if they do, typically require significantly less than face-to-face psychotherapeutic interventions. The lower clinician time and associated cost make self-help DMHIs easier to disseminate to the masses than face-to-face psychotherapeutic interventions. Second, self-help DMHIs can usually be immediately used anywhere the internet can reach. This fact means that the traditional geographic barriers that limit access to trained mental health providers can be partially overcome [5]. Third, self-help DMHIs offer the promise of addressing stigma-related barriers that may prohibit help-seeking from some people who are uncomfortable meeting with health care professionals. For example, research suggests that younger populations, including university students and especially young men, are more likely to seek online mental health support than see a mental health care professional, as it is considered to be less stigmatizing and more anonymous [7,8]. Finally, generations of people who have been exposed to the internet from childhood are now in adulthood and may be acclimatized to meeting an array of needs (eg, shopping, entertainment, socializing, and health care) via web-based applications. It therefore seems natural that some would want to meet their mental health needs in the same way. For these reasons, self-help DMHIs appear to be a clear and inevitable current and next step for mental health intervention.

The apparent potential of self-help DMHIs has grown since the onset of the COVID-19 pandemic, which has further amplified the need for low-cost, virtually delivered interventions. Rates of depression, anxiety, substance use disorder, and posttraumatic stress rose throughout the pandemic [9-11], placing an added burden on health care systems that were already strained under the weight of the virus and its sequelae. Mental health interventions that can be delivered with minimal burden to the health care system were even more sorely needed. Moreover, social distancing precautions limited the accessibility of mental health care providers in face-to-face contexts, resulting in a significant uptake in virtual or remote-delivered care. Although these changes may be partially reverting back as the pandemic recedes in public consciousness, much of the shift to virtual mental health care has endured and is expected to continue [12]. For all of these reasons, self-help DMHIs may be “the future” of mental health, if not its present.

### Let’s Make It a Big Party: Heavy Investment in Self-Help DMHIs

Widespread belief in the promise of self-help DMHIs is demonstrated by the exorbitant time, money, resources, and intellect that has been invested in their development. The number of peer-reviewed academic publications related to self-help DMHIs more than doubled in the 10 years from 2012-2021 compared to over the previous 10 years using the following search terms on PsycInfo in October 2022: noft((online or internet or web)) AND noft(psychotherap* or intervention or treatment or therap*) AND noft(self-help or self-guided). NOFT = anywhere except full text. These initiatives have also been backed by substantial funding. For example, our review of the National Institutes of Health RePORTER funding award database [13] found that over US $27.5 billion in grant funding across 66,201 projects was allocated to the study of self-help DMHIs in the fiscal years between 2012 and 2022, using the following search terms: limited to the following agencies: National Institute of Mental Health, National Institute on Alcohol Abuse and Alcoholism, Eunice Kennedy Shriver National Institute of Child Health and Human Development, and National Institute on Drug Abuse) on the National Institutes of Health RePORTER in August 2022: (online or remote or virtual or web-based or internet) AND (self-help or self help or self-management or self management or self-guided or self guided or self-directed or self directed).

However, clinical researchers are not alone in recognizing the promise and potential profit of self-help DMHIs. A 2021 report that analyzed market trends and investments from 2017 to 2020 suggested that the market value of mental health-related apps was US $5.2 billion, and one forecast predicted a compound annual growth rate of 16.5% from 2022 to 2030, bringing the forecasted revenue to US $17.5 billion by 2030 [14,15]. Clearly, self-help DMHIs have been recognized as not only potentially beneficial but also highly profitable. These findings document that there has been a great deal of attention, money, time, and intellect from academic, private, and industrial spheres devoted to the development of self-help DMHIs, at least in a North American context. It is possible that such a surge of financial interest in other areas of the world is more or less apparent than this, and research investigating whether a region of the world moderates the relative increase in funding for self-help DMHI work in recent years is needed.

### The Party Is Starting, but Where Are the Guests? Do People Actually Want Self-Help DMHIs?

The findings reviewed above suggest that the promise of self-help DMHIs is obvious to experts and investors. In fact, such promise is so seemingly obvious that many of the assumptions underpinning it may not have been adequately tested. Indeed, the notion that self-help DMHIs are actually desirable and feasible to deliver to people is an empirical question whose answer is not yet clear.

Service users consider immediate access and low wait times to be important when receiving treatment for mental health. While users expect face-to-face therapies to meet their needs on most of these factors and demonstrate a greater preference for traditional services over self-help DMHIs [16-19], self-help DMHIs are generally perceived as more convenient and accessible than traditional methods of therapy [17]. Further, potential consumers are also aligned with experts in viewing several additional potential benefits of self-help DMHIs, such as lower costs, reduced embarrassment or stigma, and avoiding wait-times, as well as other benefits such as the ability to track progress and interactivity [20]. Some have argued that such perceived advantages of self-help DMHIs may persuade users to engage with them [21] and, indeed, preferences for self-help DMHIs relative to traditional services increase when people are asked to consider the long wait times for the latter [18].

However, the perceived benefits of self-help DMHIs may not translate to their actual use. Research demonstrates that the proportion of users stating that they would likely use a self-help DMHI for their mental health is significantly greater than the relative proportion of the same users who report having a preference for it over traditional services, or an intention to actually use a self-help DMHI when given other choices [16,22,23]. Therefore, there may be a discrepancy between people’s hypothetical interest in self-help DMHIs, and their actual likelihood of engaging with it in their real lives. Such a discrepancy may be accounted for by negative perceptions of self-help DMHIs. For example, individuals perceive self-help DMHIs to be less credible relative to face-to-face interventions [17,24]. Further, while consumers recognize potential benefits of self-help DMHIs, they also have several concerns. In one study of adolescents, over half of the sample considered several perceived characteristics of self-help DMHIs to be moderately problematic, including not being able to ask questions, information feeling too general, lacking therapist support, and privacy concerns [20]. Finally, while self-help DMHIs may have the potential to reach people with elevated self-stigma related to their mental health, research suggests that, as with face-to-face interventions [25], self-stigma predicts a lower likelihood of engaging with self-help DMHIs [26].

In summary, although self-help DMHIs are widely perceived as more accessible than face-to-face mental health services, it is not yet clear whether such a perception will lead to their use. Research on the desirability of self-help DMHIs suggests that people generally do not perceive them as comparably desirable or credible, and appear less likely to use them, relative to face-to-face services. However, this body of research is small, especially compared to the relative body of self-help DMHI intervention research.

### Desirability of Self-Help DMHIs in Clinical Trial Research

In order to further assess potential desirability issues in self-help DMHIs, our team conducted a review of self-help DMHI trials registered on ClinicalTrials.gov from the years 2016-2021 to determine (1) what percentage of self-help DMHI trials met recruitment number targets and (2) when studies did not meet recruitment targets, the extent to which they fell short. Although answers to these questions do not directly test desirability, they may indicate whether researchers tend to have a more difficult time engaging users in self-help DMHIs than they initially anticipated. Also, we note that ClinicalTrials.gov is a US-based website that particularly houses registered trials from North American researchers. We selected this website as it is a particularly well-established and large database for clinical trials. However, while a review of all potential global registration sources was beyond the scope of this paper, we acknowledge that these results may not be generalizable to other regions of the world. We encourage further research into whether underrecruiting issues for self-help DMHIs generalize to other world regions.

**Figure 1:**
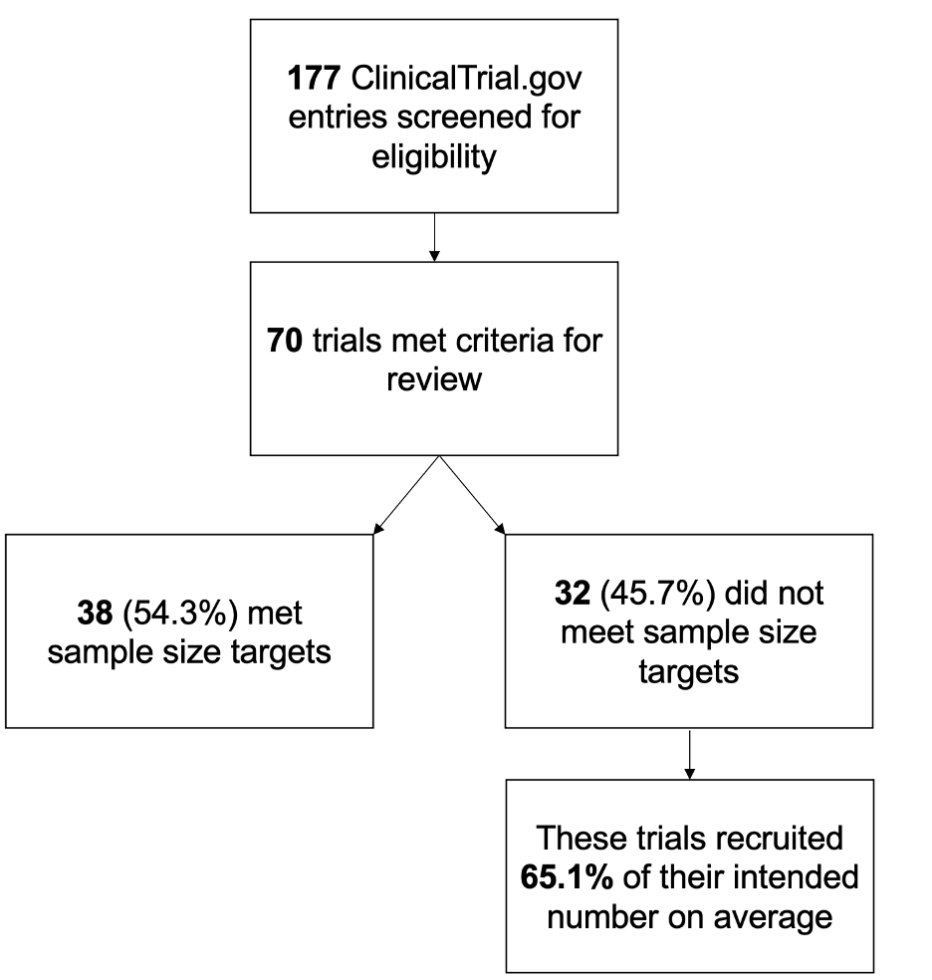
Visual representation of the number of completed self-help DMHI trials registered on ClinicalTrials.gov that met recruitment targets. Note: Of the trials that did not meet their recruitment targets, the percentage of their intended sample size recruited ranged from 14.4% to 96%. DMHI: digital mental health intervention. Note: Of the trials did that did not meet their recruitment targets, the percentage of their intended sample size recruited ranged from 14.4% to 96%.

On ClinicalTrials.gov, we used the search following terms: (“online” OR “remote” OR “web-based” OR “virtual” OR “internet”) AND (“self-help” OR “self-management” OR “self-guided” OR “self-directed”). The search focused on trials in the United States only and the study had to be marked as “completed.” Search results were then reviewed by one of the authors to determine if they met the criteria for a self-help DMHI. Uncertain classifications were presented to a group of six authors and decided by joint agreement. We referred to the earliest version of the record to determine the intended sample size and the most recent version of the record for the final sample size. We verified the final sample size through a Google Scholar search for publications linked to the clinical trials identifier. Figure 1 presents a visual representation of our findings. In total, 177 records were found, 70 of which met the criteria as a self-help DMHI. Of those 70 trials, 38 (54.3%) successfully recruited the planned sample size originally intended (the average target sample size was 220 individuals, SD 302). Of the 32 trials that did not meet recruitment targets, they recruited 65.1% of their intended sample on average.

Notably, this search did not account for trials that extended the recruitment period past the initially planned end date (eg, due to recruitment difficulties), so it may underestimate recruitment challenges. However, these findings indicate that trialists’ original estimates of the number of people who will be interested in, eligible for, and initiate participation in, the self-help DMHIs that they are testing seem to be consistently inflated. Certainly, any clinical trial failing to meet its recruitment targets is common, whether a self-help DMHI or not. Moreover, as we did not gather recruitment statistics for registered psychological intervention trials that are not DMHIs, it is difficult to clearly compare these recruitment outcomes with in-person psychotherapy research. Indeed, it is possible that DMHIs have larger recruitment targets (anticipating smaller effect sizes) which drives recruitment issues, rather than poor desirability of the intervention itself. However, unlike in-person psychotherapy trials, self-help DMHIs can often have a much broader catchment area due to not requiring participants to come into a laboratory or clinic and being able to recruit beyond the states or provinces wherein a mental health care provider is licensed [27,28]. Such consistent underrecruiting, even given these advantages, may indicate that self-help DMHIs are not as desirable as interventionists assume. These findings are concerning in a context wherein so many self-help DMHIs have already been built, tested, and are ready to be delivered.

### Why Might People Not Want Self-Help DMHIs, and What Can We Do About It?

#### Overview

Our review of clinical trials, in combination with previously reviewed research, suggests that self-help DMHIs may have a desirability issue. If self-help DMHIs are to be the solution to global difficulties disseminating mental health intervention to the vast numbers of people who need them, then it is critical that people actually want and use them. Below we outline two key potential problems that may be interfering with the uptake of self-help DMHIs, as well as potential solutions. Some of these solutions include implementation considerations that offer process support around the intervention itself and can strengthen uptake and retention. This list is not exhaustive but is intended to facilitate early anticipation of potential desirability issues in the self-help DMHI field and generate potential solutions so that the promise of these interventions may be realized.

#### Problem 1: People Are Going to Someone Else’s Party

As previously discussed, the self-help DMHI marketplace is becoming increasingly saturated, which may limit the desirability and perceived credibility of individual self-help DMHIs. Most critically, it may limit the desirability of self-help DMHIs that are evidence-based. Many technology companies that have produced self-help DMHI products have a wealth of experience in building attractive and appealing products, backed by experts in marketing, engineering, user experience, gamification, and web design. However, the extent to which mental health experts are involved in these products is unclear and, consequently, the extent to which many self-help DMHI offerings are evidence-based and likely to be efficacious is also mixed [29]. Unfortunately, consumers lack education regarding what interventions are or are not evidence-based. Indeed, Becker et al [30] found that only 2 out of 53 mental health care consumers had ever heard of the term “evidence-based practice,” and only 20% of community members can accurately define evidence-based mental health care when asked [31]. Further, studies suggest that individuals have misconceptions about the characteristics of evidence-based practice, most commonly believing that such interventions are inflexible or not person-centered [30,32,33]. Based on this research, there is a clear lack of knowledge in public consciousness regarding evidence-based and empirically supported mental health care practices, which may lead individuals to take up self-help DMHIs based on other factors. Branding, marketing, and user experience (UX) may carry a disproportionate amount of weight in determining which services are taken up by consumers, but do not necessarily translate to the extent to which those services will be efficacious. Further, grant-funded clinical researchers best positioned to produce evidence-based tools may have a small fraction of the financial and human resources to invest in increasing the attractiveness of a product (eg, marketing and web design) compared with large technology companies. Consequently, evidence-based self-help DMHIs may get lost amidst an array of offerings, with several more seemingly attractive (but potentially not evidence-based) options “soaking up all of the oxygen.” This vast competition for consumers in the self-help DMHI space may dilute the desirability of evidence-based interventions, many of which cannot compete in their capacity to draw in consumers. Moreover, individuals or people they know may try popular but not evidence-based self-help DMHIs with little benefit. Consequently, they may conclude that all self-help DMHIs are unlikely to be helpful, damaging the perceived credibility and desirability of evidence-based offerings.

#### Potential Solutions to Problem 1

Increasing the perceived credibility of self-help DMHIs, and consumers’ capacity to discern between them, requires a shift in public education tactics related to mental health. In recent years, public mental health awareness campaigns have sought to reduce stigma related to mental illness by encouraging people to talk publicly and openly about their psychological struggles and seek help in the form of medication or psychotherapy if needed (eg, Bell Let’s Talk, Jansport’s #LightentheLoad, Instagram’s #HereforYou, Maybelline New York—Brave Together). Undoubtedly, combating mental health stigma is critical in helping people gain access to the interventions they may need. However, broadly encouraging people to pursue psychiatric and psychological interventions may inadvertently mislead the public because there is a massive deficit in the availability of mental health interventions. For example, in the province that several of the authors live in, waitlists for publicly-funded psychotherapies were up to 2.5 years for youths and children in 2020 [34]. Although helping the public desire psychotherapy may improve the uptake of psychological interventions generally, there are not nearly enough mental health care providers to actually meet the demand that the destigmatizing campaigns seek to foment [5]. Therefore, a shift in public framing from solely seeking mental health support through psychiatric or individual psychotherapy interventions to include other supports across the intervention spectrum (eg, peer support, psychoeducation, and mental health literacy) is needed. Increasing awareness about the efficacy of self-help DMHIs as part of these public destigmatizing efforts is critical. Indeed, greater knowledge about self-help DMHIs is also associated with greater perceived helpfulness of them [35].

Second, awareness campaigns that encourage people to seek support from any mental health care provider or self-help DMHI are insufficient. Therefore, in addition to public efforts to encourage help-seeking generally, significant education regarding evidence-based mental health practices, empirically supported interventions, how efficacious self-help DMHIs work, and how consumers can determine the extent to which the self-help DMHI that they are considering is evidence-based are critical. Indeed, participants who receive more information about self-help DMHIs are more likely to engage in these programs in the future [36]. However, currently, participants report having little to no knowledge about them [36]. Moreover, when designing informational materials, co-creation with those who have lived experience increases the relevancy of those materials and can motivate consumers to seek out additional information about the subject in question [37].

Third, the first and second solutions mentioned earlier shift the burden of determining which interventions will be safe and efficacious to the consumer. It is also possible for self-help DMHI products to be regulated by governmental bodies. For example, work is being undertaken in the United Kingdom to evaluate factors that should be considered when regulating mental health apps [38], and at least some mental health apps in the United States have received review and approval as medical devices by the Food and Drug Administration [39]. More efforts on behalf of regulatory bodies are needed to protect the public from self-help DMHIs that may be harmful or unhelpful and identify ones that may be efficacious.

Fourth, clinical researchers seeking to construct self-help DMHIs derived from evidence-based practices or treatments may need support in constructing platforms that can compete with attractive, well-financed, but not empirically supported ones. This can therefore be an opportunity for interdisciplinary thinking and collaboration alongside professionals in other sectors such as marketing, UX, user interface design, and gamification. Those seeking grants to support the development of self-help DMHIs are advised to build salaries for such experts into their budgets or to collaborate with existing self-help DMHI developers to leverage and customize existing platforms to save costs. Moreover, funders may require education regarding why such personnel are essential to the successful building and delivery of self-help DMHIs.

Fifth, data suggests that there are some modifiable factors that can make a self-help DMHI more desirable to consumers. For example, consumers report a preference for online programs that offer brief, spaced-out content sessions instead of fewer, longer ones. Some studies suggest that they also tend to prefer self-help DMHIs that use text and images to deliver key concepts instead of video [16]. However, such findings may be moderated by the intended audience, as research with youths suggests that they prefer dynamic, interactive content to static information (eg, text) [40], and a study in a German sample generally suggested that the mode of delivery (eg, text, audio, video, or game) of a mental health intervention was not perceived to be particularly important relative to other factors [41]. Reviewing and conducting extensive desirability research about specific elements of self-help DMHIs with one’s intended audience to identify what would be appealing, attractive, and helpful to them may help researchers design more engaging tools. Further, it can be helpful to learn from those with lived experience regarding what might act as a barrier to uptake to inform the design of the self-help DMHI and identify if there are additional elements that would support its access and use [42]. Grant budgets also need to allocate funds to compensate these individuals for their time.

#### Problem 2: Guests Stop by the Party, but They Don’t Stick Around

In addition to working to initially attract people to self-help DMHIs, issues also arise in retaining them. Rates of retention in self-help DMHIs vary widely across studies. For instance, recent reviews found that retention levels ranged from 40% to 100% for mental health self-help DMHIs [43] and from 14% to 96% for wellness self-help DMHIs [44]. It is important to note that retention is measured differently across studies (eg, completing all vs half of the intervention modules) [44]. However, real-world engagement of self-help DMHI apps from user uptake data is reportedly lower than in controlled studies of similar interventions [45]. Indeed, the median retention rates for Android self-help DMHI apps after 15 and 30 days is between 3% and 4% [46], suggesting that participants may be less likely to engage in longer interventions, especially in self-paced contexts where progress is not consistently monitored as it is in research studies. Indeed, perhaps the benefits of self-help DMHIs in being accessible and easy to sign up for may also translate to issues with their capacity to retain participants, as they may require less motivation and commitment to pursue than standard face-to-face interventions. This lack of continued engagement in a self-help DMHI may hamper its efficacy and, in turn, damage the credibility and desirability of future self-help DMHIs.

#### Potential Solutions to Problem 2

To our knowledge, little attention has been paid to ways to retain, versus engage, users in self-help DMHIs. Indeed, self-help DMHIs that are built with the predominant goal of generating revenue may be incentivized to focus on getting users to buy or sign up for them, but not actually to use them. Although some studies have examined which individual characteristics predict self-help DMHI retention [47-49], few have directly tested methods to enhance it. Wojtowicz et al [49] showed that receiving phone-based coaching, compared to email-based coaching, predicted completion of modules in one online program. Similarly, self-help DMHIs incorporating human feedback and in-app mood monitoring have lower dropout rates than those that do not [50]. These studies suggest that some human involvement alongside self-help DMHIs, in addition to progress monitoring, may enhance retention. A review of the implementation literature could also offer a helpful starting point for factors that can influence retention. For example, research in telehealth interventions has demonstrated the need to ensure the fit or appropriateness of consumer-defined needs, consumer skills and capacity, and the structure of their daily lives [51]. The applicability of these factors and others noted as important for retention in the implementation literature, could be readily applied to and tested with self-help DMHIs.

In addition to these features, the content of the intervention itself may promote or reduce engagement. Geraghty et al [52] compared participants who received a self-help DMHI for body dissatisfaction containing therapeutic content alone (ie, cognitive behavioral techniques of self-monitoring and cognitive restructuring) to those who received therapeutic content within the format of a gratitude intervention. Participants enrolled in the gratitude intervention were two times more likely to complete the intervention compared to those who completed the intervention containing only therapeutic content. More research is needed to identify which forms of content may be optimal for enhancing engagement across self-help DMHIs and why.

In addition, it is well documented in the psychotherapy literature that individuals’ motivation and readiness for change informs treatment outcomes [53] and the psychotherapy landscape offers a range of tools that can increase readiness to change (eg, motivational interviewing) [54]. If the ease of signing up for self-help DMHIs means that they are able to initially attract users who vary in their motivation for change, then self-help DMHIs present both unique problems and opportunities. While they may be more likely to “lose” users who exhibited low motivation for change, they may also be uniquely poised to initially engage these users in the first place. Designing self-help DMHIs based on the premise that users may not be motivated to engage in them, and deliberately introducing interventions early with the sole purpose of increasing motivation, may be useful in promoting retention. More testing on factors that can increase motivation to engage with self-help DMHIs is therefore needed.

Moreover, technical elements of self-help DMHIs may be important to promoting user engagement and retention. People have reported that interventions with gamification features and interactive content are more enjoyable, while standard psychoeducation and visually unappealing interfaces with technological glitches are unsurprisingly rated as less attractive [55]. Therefore, the few studies that exist suggest that there are several key humans, content, and technical features that can be incorporated into self-help DMHIs to enhance their capacity to engage and retain. However, many empirical questions remain regarding best practices in promoting continued engagement in self-help DMHIs, and realizing their promise requires research to this end.

Finally, the aforementioned solutions are predicated on the notion that people should be retained. Indeed, burgeoning literature has instead focused on the development and testing of interventions that are designed without a presumption of repeat engagement (ie, single-session interventions) [56-61]. Single-session interventions overcome access issues by limiting the amount of time a mental health care provider is required to spend on one client (ie, one session vs several) and can be delivered over the internet [59], expanding their access to geographically remote or otherwise difficult-to-reach groups. Both self-administered, digital, and self-help digital single-session interventions have been developed [59-64]. Indeed, in a meta-analysis, while therapist-delivered single-session interventions yielded a larger effect size on youth psychological outcomes than self-help ones, this difference was not statistically significant [56]. More single-session approaches could be applied to the self-help DMHI landscape to address retention issues. Moreover, online single-session interventions exemplify another strategy of expanding access to mental health care which may complement self-help DMHI efforts.

## Conclusions

The mental health field faces a crisis in access to the psychotherapeutic interventions that have been developing within it for decades. While self-help DMHIs have the potential to be part of an effective response to this crisis, their promise is not fully realized because relatively little attention has been paid to whether and how consumers actually want them compared to the exorbitant amount of time, money, and labor that has been devoted to creating them. It is as if the self-help DMHI field has gone to great lengths and expense to throw a large party; venues have been booked, fancy cakes have been ordered, and entertainment has been scheduled, but nobody asked whether people can get to the venue, what food they want to or can eat, and what entertainment they like. Moreover, nobody collected RSVPs (Répondez s'il vous plaît). Is everyone going to show up?

This commentary is not intended to discourage the pursuit of self-help DMHI development and testing, but rather to encourage researchers to first establish a foundation of work required to make them successful in the long-term. This involves studying whether, when, and how self-help DMHIs are desirable to target populations, and working with experts in other content areas (eg, those with lived experience, marketing experts, UX experts, and implementation scientists and practitioners) to enhance their engagement, desirability, and retention. It also involves a deliberate shift in public education efforts to delineate evidence-based self-help DMHIs from other offerings that are currently saturating the marketplace but may not be helpful. Ultimately, with a more deliberate, thoughtful approach to self-help DMHI development and testing that carefully considers issues of desirability, engagement, and retention, we believe that self-help DMHIs may fully realize their promise as leading “the future” of mental health intervention.

## Abbreviations

DMHI: digital mental health intervention
RSVP: Répondez s'il vous plaît
UX: user experience
